# Meta-analysis of the effect of laparoscopic surgery and open surgery on long-term quality of life in patients with colorectal cancer

**DOI:** 10.1097/MD.0000000000034922

**Published:** 2023-09-08

**Authors:** Mengfan Cui, Shimin Liu

**Affiliations:** a Shanghai University of Traditional Chinese Medicine, Shanghai, China.

**Keywords:** colon cancer, laparoscopy, meta-analysis, open surgery, quality of life

## Abstract

**Objective:**

To compare the effect of laparoscopic surgery and open surgery on the quality of life of patients with colorectal cancer (CRC) in the growth period after the operation, and to provide a reference for surgical treatment decisions of patients with CRC.

**Methods:**

PubMed/MEDLINE, EMBASE, Web of Science, and Cochrane databases were searched through May 7, 2022 for clinical studies comparing the postoperative quality of life in CRC patients who underwent laparoscopic surgery with those who underwent open surgery. Data were extracted from eligible studies following rigorous quality review. All studies included patient numbers, surgery type, follow-up length, and quality of life scores.

**Results:**

A total of 6 studies were included, resulting in significantly better physical functioning scores with laparoscopic versus open surgery. (Standardized mean difference = 0.45; 95% CI (0.15, 0.75), *P* = .003). However, in general health, social functioning, bodily pain, vitality, quality of life index, Global Quality Scale, physical component summary and mental component summary, there was no telling difference between the 2 surgical therapies.

**Conclusion:**

Compared with open surgery, laparoscopic surgery has weak advantages. There was no noteworthy difference in the long-term quality of life between the 2 surgical treatments for CRC patients. Whether laparoscopic surgery can bring more improvement to the quality of life of patients with CRC needs more high-quality clinical randomized studies to verify.

## 1. Introduction

Colorectal cancer (CRC) is the third most common cancer worldwide and the second leading cause of cancer-related mortality.^[1]^ Factors such as eating habits, familial inheritance, overweight, and smoking increase the risk of CRC.^[2–4]^ Surgical resection is usually the mainstay of treatment in patients with CRC.^[5]^ Radical open surgery (OS) treatment has been the main treatment until 1983 when laparoscopic surgery was first applied to appendectomy,^[6]^ and laparoscopic surgery has since been widely used in abdominal surgery. The efficacy of laparoscopic surgery was not significantly different from that of open surgery.^[7,8]^ In addition, patients with smaller wounds, less bleeding, and a faster recovery rate are reasons for the rapid clinical use and promotion of laparoscopy.^[9]^ Laparoscopic surgery can also avoid some serious postoperative complications, such as venous thrombosis and severe anastomotic leakage.^[10,11]^ In addition, to some extent, laparoscopic surgery can reduce the cost of surgery and nursing care for patients and improve the efficiency of the use of medical resources.^[12–14]^

At present, laparoscopic surgery or laparoscopic-assisted surgical therapy has become the recommended treatment for patients with CRC.^[15]^ Better tumor resection and fewer side effects have been recognized. However, it is unknown whether laparoscopic surgery has such great advantages in improving the quality of life.^[16,17]^ Long-term quality of life is also an important outcome measure for CRC patients.^[18]^ It has been pointed out that different treatment options can cause long-term distress to postoperative complications and quality of life in cancer patients.^[19]^ This article focuses on the quality evaluation and meta-analysis of the impact of laparoscopic surgery and open surgery on the quality of life of patients with CRC, to explore which surgical therapy is more suitable for patients with CRC, and to contribute to the improvement of the quality of life of patients, to better guide the development of clinical treatment options for patients with CRC.

## 2. Materials and methods

### 2.1. Literature search

A comprehensive electronic literature search of PubMed/MEDLINE, EMBASE, Web of Science, and Cochrane databases and conference proceedings was performed to identify eligible articles from the start of indexing to May 7, 2022, without language restriction. The following text words and medical subject headings were used for the search: “colectomy,” “colectomies,” “hemicolectomy,” “colonic Neoplasms,” “Colon Cancers,” “Quality of Life,” “HRQOL.” The work has been reported in line with Preferred Reporting Items for Systematic Reviews and Meta-Analyses guidelines.^[20]^

### 2.2. Study option

Two reviewers independently completed eligibility assessments and screened records identified in the initial search.

1. Inclusion criteria were: the study population must be 18 years of age and meet the diagnostic criteria for CRC^[3]^; the study included both open surgery and laparoscopy; the study report included the quality of life after open surgery and laparoscopy; the article has clear criteria for the evaluation of the quality of life.
2. Exclusion criteria were: repeated publications; reviews, conference abstracts, case reports, and animal trials; lack of follow-up time or serious data dropouts.

### 2.3. Data extraction

Two researchers extracted data from each study and entered it into a database. The following details were extracted from each study: general study characteristics (first author, year, type of study), clinical characteristics (age, number of patients, location of CRC, cancer type, follow-up time), and outcome measures (type of surgery, quality of life measures).

### 2.4. Quality evaluation of methodology

The quality of studies included in the studies was evaluated using the bias analysis assessment tool provided by Cochrane Handbook for Systematic Reviews of Interventions 6.2. This evaluation includes 7 aspects: random sequence generation (selective bias), allocation concealment (selective bias), blinding of implementers and participants (implementation bias), blinding of outcome assessors (observation bias), the integrity of data results (follow-up bias), selective reporting of study results (reporting bias), and other sources of bias. Each included study is evaluated item by item according to the above criteria. If the original study completely meets the above criteria, its quality is “low risk”, indicating the overall low risk of bias, and the study quality is high; if the original study partially meets the above criteria, its quality is “unclear risk,” indicating that the possibility of bias is moderate; if the original study completely fails to meet the above criteria, its quality is “high risk,” indicating that there is a high risk of bias, indicating that the study quality is low.

### 2.5. Statistical analysis

In this meta-analysis, continuous variables were analyzed with the standardized mean difference (SMD) and expressed with 95% CIs. Fixed effects models were used to determine the purpose of comparison between laparoscopic surgery and open surgery, and heterogeneity tests were performed on the included articles. If I^2^ ≤50%, it indicated that the homogeneity of the included studies was good, and a fixed effects model was used; if I^2^ >50%, it was considered that there was heterogeneity between the included studies, and a random effects model was used. Sensitivity analysis of sources of heterogeneity was performed by removing each article. The funnel plot method (Review Manager 5.4) was used to test publication bias. *P* value < .05 was considered statistically significant

## 3. Results

### 3.1. Literature search results and study characteristics

**Figure 1.**
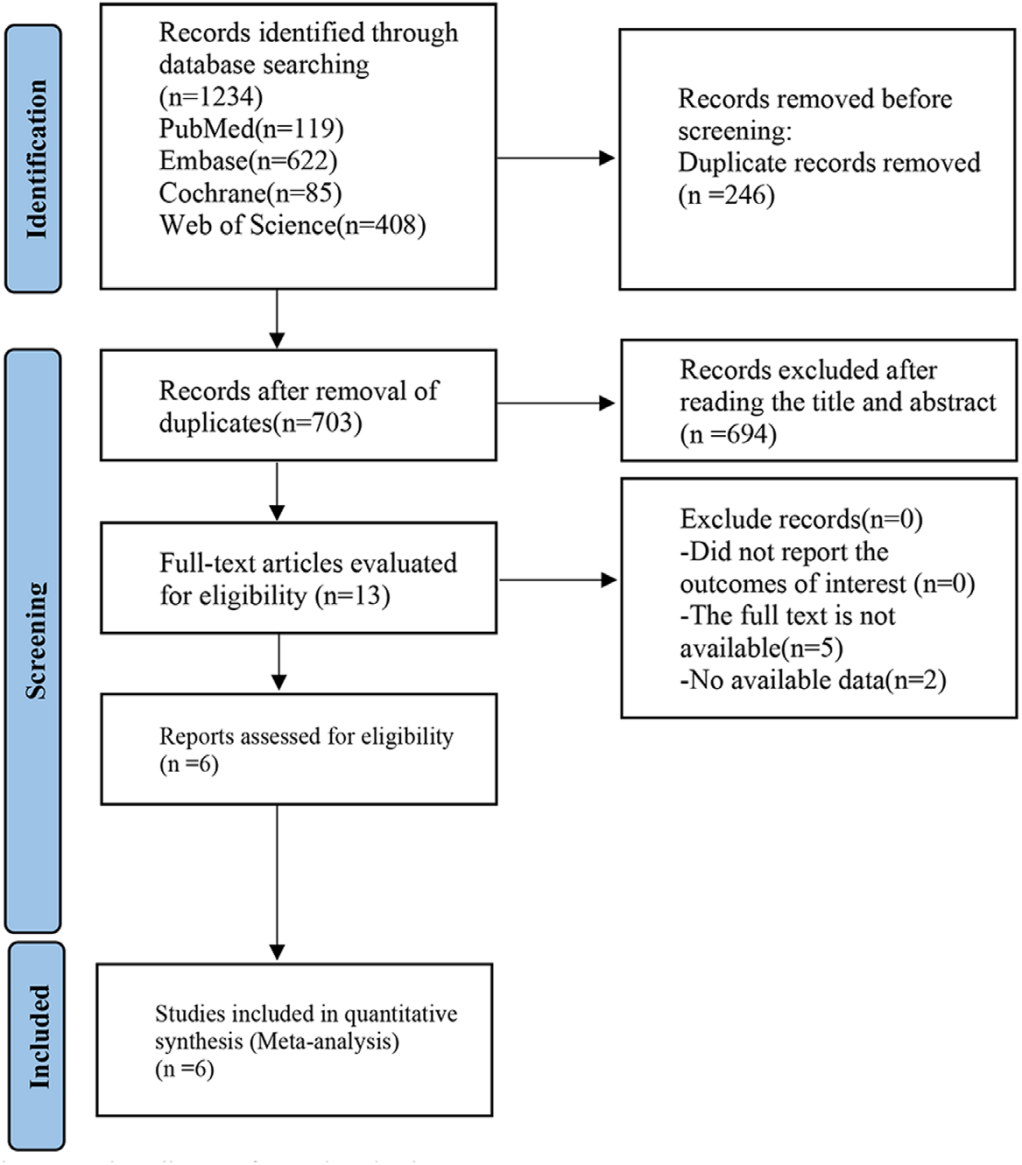
Flow diagram for study selection process.

Through a systematic search of PubMed/MEDLINE, EMBASE, Web of Science, and Cochrane databases, 1234 potentially relevant literature were preliminarily retrieved, 246 pieces of repeated literature were excluded, and 6 pieces of literature inconsistent with study content were removed from reading titles and abstracts, resulting in the last 13 literature. After reading the full text, 2 articles were excluded because they did not provide the data required for inclusion, 5 articles were excluded because the study type was not a randomized controlled study design, and finally 6 studies were included.^[20–25]^ Literature screening procedures and results are shown in Figure 1.

### 3.2. Methodological quality evaluation of included studies

**Figure 2.**
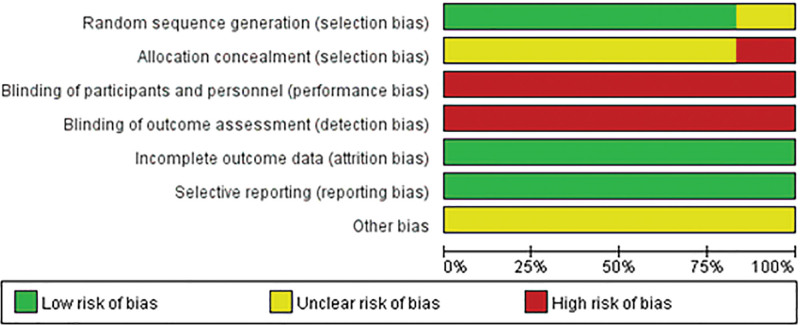
Assessment of risk of bias in included studies.

All 4 included studies^[20–25]^ clearly described the method of randomization sequence generation, the concealment of the allocation scheme, and other bias were shown “unclear.” Both blinding of participants and personnel and blinding of outcome assessment were “high risk of bias.” All 4 studies achieved complete and selective reporting of study results. The proportions of each item of methodological quality evaluation of the included studies are shown in Figure 2. The results of the NOS quality evaluation for the 2 studies that were not randomized controlled studies are summarized in Table 1.

**Table 1:** Characteristics of the studies included in the systematic review and meta-analysis.

Study	Country	Type of study	Age		Number of participants		BMI (kg/m^2^)		Location of tumor	Stage	Follow-up (mo)	Outcome	NOS scores
			LA	OC	LA	OC	LA	OC					
Braga, 2005	Italy	RCT	65 ± 13	67 ± 11	190	201	N/A	N/A	Right colectomy	I–IV	48	F1; F2; F3; F4	/
Fuji, 2010	Japan	Case-Matched	64.8 ± 9.8	65.8 ± 10.5	258	258	22.6 ± 3.5	23.1 ± 3.6	Left-sided colectomy	II–IV	62	F1; F2; F3; F4; F5; F6; F7; F8; F9; F10	7/9
Ihnát, 2014	Czech Republic	Cohort	60.4 ± 11.7	63.8 ± 9.3	83	65	26.2 ± 10.6	24.7 ± 13.9	Anterior resection of rectum	I–III	6	F2; F5; F6; F8; F9; F10	8/9
McCombie, 2018	New Zealand	RCT	70.62 ± 9.97	69.13 ± 11.38	180	213	71.04 ± 17.19	72.75 ± 15.10	right	I–III	2	F14; F15; F16	/
Stucky, 2011	America	RCT	69.5 ± 10.59	68.2 ± 11.84	219	230	NR	NR	NR	I–IV	18	F16; F17; F18; F19; F20; F21; F22; F23; F24; F25	/
Weeks, 2002	America	RCT	68.2 ± 17	69.4 ± 38.95	228	221	NR	NR	NR	I–IV	2	F15; F16; F24	/

N/A = not applicable, NR = not reported, RCT = Randomized controlled trial, LA = laproscopic surgery, OC = open coloctomy; F1 = general health; F2 = physical functioning; F3 = social functioning; F4 = DFS; F5 = bodily pain; F6 = vitality; F7 = role emotional; F8 = mental health; F9 = PCS physical component summary; F10 = MCS mental component summary; F14 = Global QoL; F15 = SDS Symptoms distress scale; F16 = QLI quality of life index; F17 = nausea and vomiting; F18 = bowel function; F19 = concentration; F20 = cough; F21 = outlook; F22 = daily living; F23 = health; F24 = global rating scale; F25 = pain frequency.

### 3.3. Study characteristics

We focused on the sample size of the included studies, the length of follow-up (with the most recent follow-up as the outcome), and the questionnaires and measures used to assess the quality of life. The main characteristics of eligible studies were described in detail in Table 1.

### 3.4. Quality of life in patients with CRC after surgery

Among the included literature, the most used questionnaire to evaluate the quality of life was the Short Form 36 Health Survey questionnaire (SF-36),^[21,22,24]^ and the questionnaires used in other studies included SDS, quality of life index (QLI),^[20,23,25]^ etc. We combined the 3 quality-of-life measures.

### 3.5. General health

**Figure 3.**
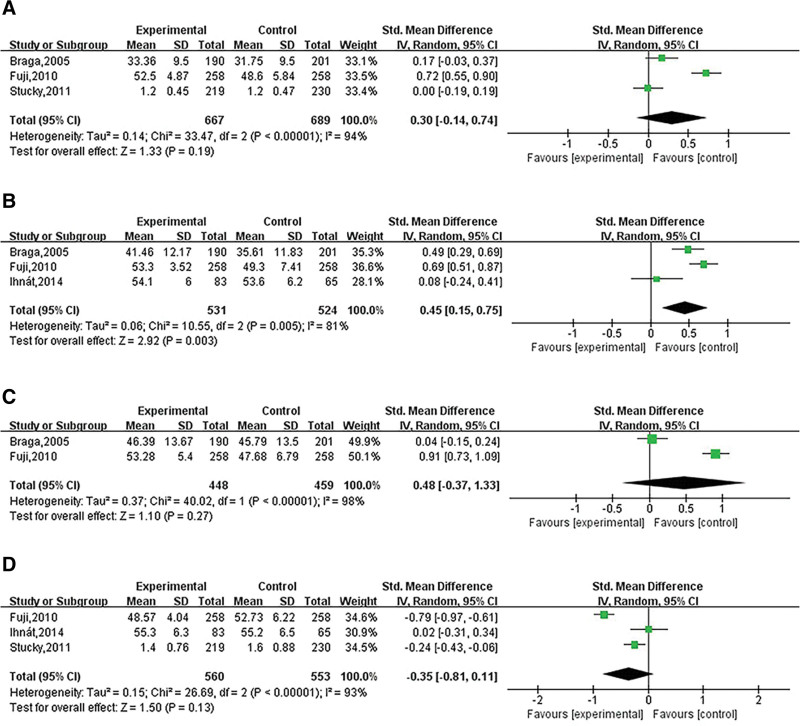
Forest plot of laparoscopic versus open colorectal surgery for (A) general health, (B) physical functioning, (C) social functioning, and (D) bodily pain.

Three articles contained general health indicators, including 667 in the laparoscopic group and 689 in the open group. The heterogeneity test showed that the heterogeneity was large (I^2^ = 94%, *P* < .00001), and the random model was used for data analysis, and the results showed that there was no noticeable difference between the 2 surgical therapies (SMD = 0.30; 95% CI [−0.14, 0.74], *P* = .19, Z = 1.33; Fig. 3A). Sensitivity was assessed by removing the included articles one by one. The arbitrary removal of any included article through sensitivity analysis did not compromise the results, indicating that the effect size pooling results were stable and reliable.

### 3.6. Physical functioning

Three articles contained physical functioning (PF) indicators, including 531 in the laparoscopic group and 524 in the open group. The heterogeneity test showed that the heterogeneity was large (I^2^ = 81%, *P* = .005), and the random model was used for data analysis. The results showed that the laparoscopic surgery group was significantly better than the open surgery group, and the difference was statistically significant (SMD = 0.45; 95% CI [0.15, 0.75] *P* = .003, Z = 2.92; Fig. 3B). Sensitivity was assessed by removing the included articles one by one. The arbitrary removal of any included article through sensitivity analysis didn’t compromise the results, indicating that the effect size pooling results were stable and reliable.

### 3.7. Social Functioning

Two articles contained social functioning (SF) measures, including 448 in the laparoscopic group and 459 in the open group. The heterogeneity test showed that the heterogeneity was large (I^2^ = 98%, *P* < .00001), and the random model was used for data analysis, and the results showed that there was no obvious difference between the 2 surgical therapies (SMD = 0.48; 95% CI [−0.37, 1.33], *P* = .27, Z = 1.10; Fig. 3C). Sensitivity was assessed by removing the included articles one by one. The arbitrary removal of any included article through sensitivity analysis didn’t compromise the results, indicating that the effect size pooling results were stable and reliable.

### 3.8. Bodily pain

Three articles contained bodily pain indicators, including 560 in the laparoscopic group and 553 in the open group. The heterogeneity test showed that the heterogeneity was large (I^2^ = 93%, *P* < .00001), and the random model was used for data analysis, and the results showed that there was no significant difference between the 2 surgical therapies (SMD = −0.35; 95% CI [−0.81, 0.11], *P* = .13, Z = 1.50; Fig. 3D). Sensitivity was assessed by removing the included articles one by one. The arbitrary removal of any included article through sensitivity analysis didn’t compromise the results, indicating that the effect size pooling results were stable and reliable.

### 3.9. Vitality

**Figure 4.**
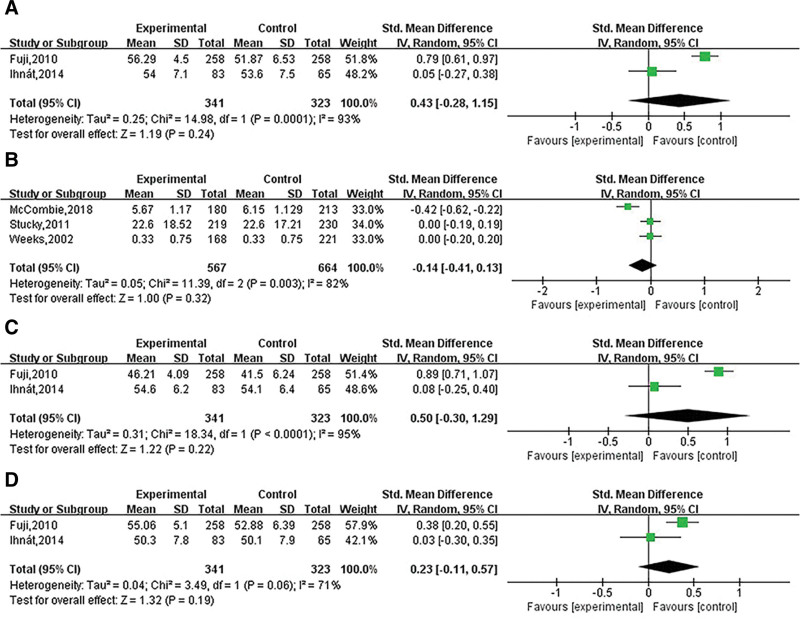
Forest plot of laparoscopic versus open colorectal surgery for (A) vitality, (B) Quality of life index (QLI), (C) physical component summary (PCS), and (D) mental component summary (MCS).

Two articles contained vitality indicators, including 341 in the laparoscopic group and 323 in the open group. The heterogeneity test showed that the heterogeneity was large (I^2^ = 93%, *P* = .0001), and the random model was used for data analysis, and the results showed that there was no telling difference between the 2 surgical therapies (SMD = 0.43; 95% CI [−0.28, 1.15], *P* = .24, Z = 1.19; Fig. 4A). Sensitivity was assessed by removing the included articles one by one. The arbitrary removal of any included article through sensitivity analysis did not compromise the results, indicating that the effect size pooling results were stable and reliable.

### 3.10. Quality of life index (QLI)

Two articles included QLI indicators, including 567 patients in the laparoscopic surgery group and 664 patients in the open surgery group. The heterogeneity test showed that the heterogeneity was large (I^2^ = 82%, *P* = .003), and the random model was used for data analysis, and the results showed that there was no obvious difference between the 2 surgical therapies (SMD = −0.14; 95% CI [−0.41, 0.13], *P* = .32, Z = 1.00; Fig. 4B). Sensitivity was assessed by removing the included articles one by one. The arbitrary removal of any included article through sensitivity analysis didn’t compromise the results, demonstrating that the effect size pooling results were stable and reliable.

### 3.11. Physical component summary

Two articles contained physical component summary indicators, including 341 in the laparoscopic group and 323 in the open group. The heterogeneity test showed that the heterogeneity was large (I^2^ = 95%, *P* = .02), and the random model was used for data analysis, and the results showed that there was no noticeable difference between the 2 surgical therapies (SMD = 0.50; 95% CI [−0.30, 1.29], *P* = .22, Z = 1.22; Fig. 4C). Sensitivity was assessed by removing the included articles one by one. The arbitrary removal of any included article through sensitivity analysis didn’t compromise the results, indicating that the effect size pooling results were stable and reliable.

### 3.12. Mental component summary

Two articles contained mental component summary indicators, including 387 in the laparoscopic group and 451 in the open group. The heterogeneity test showed that the heterogeneity was large (I^2^ = 71%, *P* = .06), and the random model was used for data analysis, and the results showed that there was no significant difference between the 2 surgical therapies (SMD = 0.23; 95% CI [−0.11, 0.57], *P* = .19, Z = 1.32; Fig. 4D). Sensitivity was assessed by removing the included articles one by one. The arbitrary removal of any included article through sensitivity analysis didn’t compromise the results, indicating that the effect size pooling results were stable and reliable.

## 4. Discussion

The choice of CRC resection is an important clinical factor affecting the quality of life of patients. This paper aimed to verify whether there was a difference in the impact of laparoscopic surgery on quality of life compared with open surgery.

Based on the results of the meta-analysis, the advantage of laparoscopic surgery over open surgery in terms of quality of life for CRC patients was very weak. In the study results, only 1 advantage of laparoscopic surgery could be observed for Physical Functioning. However, there was no statistically significant difference between laparoscopy and open surgery in the remaining 9 observed outcome measures.

PF is an indicator of the ability of daily living and PF of the study population.^[26,27]^ PF can better reflect the postoperative recovery of patients.^[28]^ Laparoscopic surgery allows patients to shorten the length of hospital stay, recover faster, and have a milder inflammatory response, allowing patients to recover faster from postoperative levels to preoperative levels.^[29–32]^ These could be the reason for the higher PF score in laparoscopic surgery. However, laparoscopic surgery has not brought more improvement to the quality of life of patients. More outcome measures did not suggest a difference in the impact of laparoscopy versus open surgery on quality of life.

As early as 2010, in a systematic review of laparoscopic versus open surgery for CRC in the Netherlands, it was concluded that there was no clinically relevant difference in the impact of the above 2 surgical therapies on quality of life.^[33]^ Guillou et al,^[34]^ through a rigorous study design, found that at 3 months after surgery, the quality of life of patients in the laparoscopic surgery group and patients in the open surgery group reached the baseline value basically, and there was no noticeable difference between the 2 surgical therapies. This is consistent with our conclusions. There was no noteworthy difference in the long-term postoperative quality of life between the 2 surgical therapies. Factors affecting the quality of life are complex. Standardized postoperative care and follow-up can improve the quality of life of patients.^[35,36]^ In addition, personality,^[37]^ and surgical wound recovery effect^[38]^ can also have an impact on the quality of life of patients. Postoperative complications are also important contributing factors.^[39,40]^ CRC patients usually face the possibility of complications such as incisional hernia, small-bowel obstruction, and Adhesion in their postoperative recovery.^[41–44]^ These made the patient endure more pain and even required a second operation to resolve.^[45]^ Therefore, surgical therapy is not the only factor affecting the quality of life.

The survival time and postoperative complications are also important contributing factors of cancer patients are also important factors affecting the quality of life.^[39,40]^ Disease-free survival has long been a key observation in cancer research, and CRC is no exception.^[41]^ There is no doubt that the extension of disease-free survival is significant for the improvement of quality of life of CRC patients. The included studies shows^[21,22]^ that the disease-free survival of patients undergoing both surgical therapies gradually decreases with the progression of the stage at 5 years. The difference caused by the effect of the 2 therapies on disease-free survival is not significant.^[20–24]^ CRC patients usually face the possibility of complications such as incisional hernia, small-bowel obstruction, and Adhesion in their postoperative recovery.^[42–44]^ These made the patient endure more pain and even required a second operation to resolve.^[45]^ Compared to open surgery, laparoscopic surgery is not perfect for CRC patients either.

Theodoropoulos et al^[46]^ came to the opposite conclusion that the use of laparoscopic surgery allows patients to have a higher postoperative quality of life than preoperative levels compared to laparotomy. However, he exercised a prospective study experiment, the sample size of the study was small, it was not a randomized controlled study design, and the reliability of the conclusions was questionable. Glaowacka et al^[47]^ also came to the idea that laparoscopic surgery could bring short-term advantages to patients, but he added that such advantages would gradually disappear in long-term outcomes. Lizdenis^[48]^ concluded that laparoscopic surgery brings more favorable conditions to the quality of life of patients with CRC in short-term follow-up results, but did not consider whether this conclusion remains true in long-term quality of life.

In addition, there are limitations to our article. The first is the insufficient number of included studies, only 6, and the lack of high-quality randomized controlled studies, causing the problem of large heterogeneity of studies. Second, the most recent of the included studies was also published 5 years ago. The results of these studies were not particularly timely. Besides, the randomization design of some studies is not rigorous enough, and the risk bias is large. Fourth, the methods to assess the postoperative quality of life are not uniform enough. The questionnaire used was different and the SF-36 was used at most. Outcome measures were also analyzed based on SF-36. How to accurately reflect differences in quality of life remains a major challenge for us.^[49]^

Enhanced recovery after surgery (ERAS) is a multimodal, multidisciplinary, coordinated approach to the treatment of surgical patients. ERAS aims to reduce perioperative stress, maintain postoperative physiological function, and improve recovery after surgery.^[50,51]^ Over the past few years, ERAS has become more common in our daily laparoscopic practice.^[52]^ It has a positive effect on the postoperative recovery of all kinds of surgery, especially CRC surgery.^[53]^ This is an additional factor that can have a direct impact on the patient quality of life.^[54]^ Considering this view, we believe that reevaluating the study in terms of whether or not to receive ERAS treatment to perform an updated literature review and a new meta-analysis would be more instructive for improving the quality of life of CRC patients.

## 5. Conclusion

Laparoscopic surgery is superior to open surgery only in terms of PF, demonstrating a slight advantage. There was no significant difference in long-term quality of life improvement between laparoscopic surgery and open surgery in patients with CRC. Evaluation of quality of life is vulnerable to multiple factors. Whether laparoscopic surgery can bring more improvement to the quality of life of patients with CRC requires more high-quality clinical randomized trials to verify.

## Acknowledgments

We would like to thank the researchers and study participants for their contributions.

## Author contributions

**Conceptualization:** Mengfan Cui.

**Data curation:** Mengfan Cui.

**Formal analysis:** Mengfan Cui.

**Funding acquisition:** Mengfan Cui.

**Investigation:** Mengfan Cui.

**Methodology:** Mengfan Cui.

**Project administration:** Shimin Liu.

**Resources:** Shimin Liu.

**Software:** Shimin Liu.

**Supervision:** Shimin Liu.

**Validation:** Shimin Liu.

**Visualization:** Shimin Liu.

**Writing – original draft:** Mengfan Cui, Shimin Liu.

**Writing – review & editing:** Mengfan Cui, Shimin Liu.

## References

[1] Keum N Giovannucci E. Global burden of colorectal cancer: emerging trends, risk factors and prevention strategies. Nat Rev Gastroenterol Hepatol. 2019;16:713–32. 31455888 10.1038/s41575-019-0189-8

[2] Jasperson KW Tuohy TM Neklason DW. Hereditary and familial colon cancer. Gastroenterology. 2010;138:2044–58. 20420945 10.1053/j.gastro.2010.01.054 PMC3057468

[3] Ligibel JA Bohlke K May AM. Exercise, diet, and weight management during cancer treatment: ASCO guideline. J Clin Oncol. 2022;40:2491–507. 35576506 10.1200/JCO.22.00687

[4] Murphy N Ward HA Jenab M. Heterogeneity of colorectal cancer risk factors by anatomical subsite in 10 European countries: a multinational cohort study. Clin Gastroenterol Hepatol. 2019;17:1323–1331.e6. 30056182 10.1016/j.cgh.2018.07.030 PMC6542674

[5] Wyld L Audisio RA Poston GJ. The evolution of cancer surgery and future perspectives. Nat Rev Clin Oncol. 2015;12:115–24. 25384943 10.1038/nrclinonc.2014.191

[6] Semm K. Endoscopic appendectomy. Endoscopy. 1983;15:59–64. 6221925 10.1055/s-2007-1021466

[7] Chi Z Li Z Cheng L. Comparison of long-term outcomes after laparoscopic-assisted and open colectomy for splenic flexure cancer. J BUON. 2018;23:322–8. 29745072

[8] Buunen M Veldkamp R Hop WC. Survival after laparoscopic surgery versus open surgery for colon cancer: long-term outcome of a randomised clinical trial. Lancet Oncol. 2009;10:44–52. 19071061 10.1016/S1470-2045(08)70310-3

[9] Veldkamp R Kuhry E Hop WC. Laparoscopic surgery versus open surgery for colon cancer: short-term outcomes of a randomised trial. Lancet Oncol. 2005;6:477–84. 15992696 10.1016/S1470-2045(05)70221-7

[10] Cheng CL Rezac C. The role of robotics in colorectal surgery. BMJ. 2018;360:j5304. 29440057 10.1136/bmj.j5304

[11] Kang J Kim H Park H. Risk factors and economic burden of postoperative anastomotic leakage related events in patients who underwent surgeries for colorectal cancer. PLoS One. 2022;17:e0267950. 35584082 10.1371/journal.pone.0267950 PMC9116683

[12] Zogg CK Najjar P Diaz AJ. Rethinking priorities: cost of complications after elective colectomy. Ann Surg. 2016;264:312–22. 26501705 10.1097/SLA.0000000000001511

[13] Kazama K Numata M Aoyama T. Laparoscopic vs. open surgery for stage II/III colon cancer patients with body mass index >25 kg/m^2^. In Vivo. 2020;34:2079–85. 32606186 10.21873/invivo.12011 PMC7439910

[14] Gianotti L Nespoli L Rocchetti S. Gut oxygenation and oxidative damage during and after laparoscopic and open left-sided colon resection: a prospective, randomized, controlled clinical trial. Surg Endosc. 2011;25:1835–43. 21136109 10.1007/s00464-010-1475-2

[15] Lai JH Law WL. Laparoscopic surgery for colorectal cancer. Br Med Bull. 2012;104:61–89. 23086860 10.1093/bmb/lds026

[16] Niu SF Cheng SY Chin CH. Quality of life and severity of symptom differences between post open colectomy and laparoscopic colectomy in colorectal cancer patients. Cancer Nurs. 2021;44:E221–8. 32132368 10.1097/NCC.0000000000000793

[17] Dowson HM Cowie AS Ballard K. Systematic review of quality of life following laparoscopic and open colorectal surgery. Colorectal Dis. 2008;10:757–68. 18573115 10.1111/j.1463-1318.2008.01603.x

[18] Arndt V Koch-Gallenkamp L Jansen L. Quality of life in long-term and very long-term cancer survivors versus population controls in Germany. Acta Oncol. 2017;56:190–7. 28055266 10.1080/0284186X.2016.1266089

[19] Hamer J McDonald R Zhang L. Quality of life (QOL) and symptom burden (SB) in patients with breast cancer. Support Care Cancer. 2017;25:409–19. 27696078 10.1007/s00520-016-3417-6

[20] McCombie AM Frizelle F Bagshaw PF. The ALCCaS trial: a randomized controlled trial comparing quality of life following laparoscopic versus open colectomy for colon cancer. Dis Colon Rectum. 2018;61:1156–62. 30192324 10.1097/DCR.0000000000001165

[21] Fujii S Ota M Ichikawa Y. Comparison of short, long-term surgical outcomes and mid-term health-related quality of life after laparoscopic and open resection for colorectal cancer: a case-matched control study. Int J Colorectal Dis. 2010;25:1311–23. 20533052 10.1007/s00384-010-0981-y

[22] Braga M Frasson M Vignali A. Laparoscopic vs. open colectomy in cancer patients: long-term complications, quality of life, and survival. Dis Colon Rectum. 2005;48:2217–23. 16228828 10.1007/s10350-005-0185-7

[23] Stucky CC Pockaj BA Novotny PJ. Long-term follow-up and individual item analysis of quality of life assessments related to laparoscopic-assisted colectomy in the COST trial 93-46-53 (INT 0146). Ann Surg Oncol. 2011;18:2422–31. 21452066 10.1245/s10434-011-1650-2 PMC3947623

[24] Ihnát P Martínek L Mitták M. Quality of life after laparoscopic and open resection of colorectal cancer. Dig Surg. 2014;31:161–8. 24992997 10.1159/000363415

[25] Weeks JC Nelson H Gelber S. Short-term quality-of-life outcomes following laparoscopic-assisted colectomy vs open colectomy for colon cancer: a randomized trial. JAMA. 2002;287:321–8. 11790211 10.1001/jama.287.3.321

[26] Korolija D Sauerland S Wood-Dauphinée S. Evaluation of quality of life after laparoscopic surgery: evidence-based guidelines of the European Association for Endoscopic Surgery. Surg Endosc. 2004;18:879–97. 15108103 10.1007/s00464-003-9263-x

[27] Ware J Kosinski M Bjorner J. User’s Manual for the SF-36v2 Health Survey. Lincoln, RI: Quality Metric. Inc. 2007.

[28] Parry SM Huang M Needham DM. Evaluating physical functioning in critical care: considerations for clinical practice and research. Crit Care. 2017;21:249. 28978333 10.1186/s13054-017-1827-6 PMC5628423

[29] Krielen P Ten Broek RPG van Dongen KW. Adhesion-related readmissions after open and laparoscopic colorectal surgery in 16 524 patients. Colorectal Dis. 2022;24:520–9. 34919765 10.1111/codi.16024

[30] Son IT Kim JY Kim MJ. Clinical and oncologic outcomes of laparoscopic versus open surgery in elderly patients with colorectal cancer: a retrospective multicenter study. Int J Clin Oncol. 2021;26:2237–45. 34453641 10.1007/s10147-021-02009-4

[31] Yang L Wang T Weidner TK. Intraoperative musculoskeletal discomfort and risk for surgeons during open and laparoscopic surgery. Surg Endosc. 2021;35:6335–43. 33083930 10.1007/s00464-020-08085-3

[32] Braga M Vignali A Zuliani W. Metabolic and functional results after laparoscopic colorectal surgery: a randomized, controlled trial. Dis Colon Rectum. 2002;45:1070–7. 12195192 10.1007/s10350-004-6362-2

[33] Bartels SA Vlug MS Ubbink DT. Quality of life after laparoscopic and open colorectal surgery: a systematic review. World J Gastroenterol. 2010;16:5035–41. 20976839 10.3748/wjg.v16.i40.5035 PMC2965279

[34] Guillou PJ Quirke P Thorpe H. Short-term endpoints of conventional versus laparoscopic-assisted surgery in patients with colorectal cancer (MRC CLASICC trial): multicentre, randomised controlled trial. Lancet. 2005;365:1718–26. 15894098 10.1016/S0140-6736(05)66545-2

[35] Wang P Chen H Ji Q. Application of operating room nursing intervention to incision infection of patients undergoing gastrointestinal surgery can reduce complications and improve gastrointestinal function. Front Surg. 2022;9:842309. 35242807 10.3389/fsurg.2022.842309 PMC8885529

[36] Duan X Su D Yu H. Adoption of Artificial Intelligence (AI)-based Computerized Tomography (CT) evaluation of comprehensive nursing in the operation room in laparoscopy-guided radical surgery of colon cancer. Comput Intell Neurosci. 2022;2022:2180788. 35300396 10.1155/2022/2180788 PMC8923753

[37] Siassi M Weiss M Hohenberger W. Personality rather than clinical variables determines quality of life after major colorectal surgery. Dis Colon Rectum. 2009;52:662–8. 19404072 10.1007/DCR.0b013e31819ecf2e

[38] Eshuis EJ Slors JF Stokkers PC. Long-term outcomes following laparoscopically assisted versus open ileocolic resection for Crohn’s disease. Br J Surg. 2010;97:563–8. 20175126 10.1002/bjs.6918

[39] Brown SR Mathew R Keding A. The impact of postoperative complications on long-term quality of life after curative colorectal cancer surgery. Ann Surg. 2014;259:916–23. 24374539 10.1097/SLA.0000000000000407

[40] Schiergens TS Hoffmann V Schobel TN. Long-term quality of life of patients with permanent end ileostomy: results of a nationwide cross-sectional survey. Dis Colon Rectum. 2017;60:51–60. 27926557 10.1097/DCR.0000000000000732

[41] Morris JS. Disease-free survival is a promising surrogate for overall survival in colorectal cancer studies. J Natl Cancer Inst. 2022;114:5–6. 34505876 10.1093/jnci/djab188 PMC8755480

[42] Van den Dop LM De Smet GHJ Kleinrensink GJ. Hybrid operation technique for incisional hernia repair: a systematic review and meta-analysis of intra- and postoperative complications. Hernia. 2021;25:1459–69. 34537886 10.1007/s10029-021-02497-3 PMC8613158

[43] Jeppesen M Tolstrup MB Gögenur I. Chronic pain, quality of life, and functional impairment after surgery due to small bowel obstruction. World J Surg. 2016;40:2091–7. 27384171 10.1007/s00268-016-3616-9

[44] Erkek AB Remzi FH Hammel JP. Effect of small bowel obstruction on functional outcome and quality of life in patients with ileal pouch-anal anastomosis: 10-year follow-up study. J Gastroenterol Hepatol. 2008;23:119–25. 18171350 10.1111/j.1440-1746.2006.04789.x

[45] Vignali A Elmore U Aleotti F. Re-laparoscopy in the treatment of anastomotic leak following laparoscopic right colectomy with intracorporeal anastomosis. Surg Endosc. 2021;35:6173–8. 33104916 10.1007/s00464-020-08113-2

[46] Theodoropoulos GE Karantanos T Stamopoulos P. Prospective evaluation of health-related quality of life after laparoscopic colectomy for cancer. Tech Coloproctol. 2013;17:27–38. 23065134 10.1007/s10151-012-0869-7

[47] Głowacka-Mrotek I Tarkowska M Nowikiewicz T. Prospective evaluation of the quality of life of patients undergoing surgery for colorectal cancer depending on the surgical technique. Int J Colorectal Dis. 2019;34:1601–10. 31396708 10.1007/s00384-019-03357-4

[48] Lizdenis P Birutis J Čelkienė I. Short-term results of quality of life for curatively treated colorectal cancer patients in Lithuania. Medicina (Kaunas). 2015;51:32–7. 25744773 10.1016/j.medici.2015.01.006

[49] Haraldstad K Wahl A Andenæs R. A systematic review of quality of life research in medicine and health sciences. Qual Life Res. 2019;28:2641–50. 31187410 10.1007/s11136-019-02214-9 PMC6761255

[50] Gustafsson UO Scott MJ Hubner M. Guidelines for perioperative care in elective colorectal surgery: enhanced recovery after surgery (ERAS(®)) society recommendations: 2018. World J Surg. 2019;43:659–95. 30426190 10.1007/s00268-018-4844-y

[51] Arrick L Mayson K Hong T. Enhanced recovery after surgery in colorectal surgery: impact of protocol adherence on patient outcomes. J Clin Anesth. 2019;55:7–12. 30583114 10.1016/j.jclinane.2018.12.034

[52] Ljungqvist O Scott M Fearon KC. Enhanced recovery after surgery: a review. JAMA Surg. 2017;152:292–8. 28097305 10.1001/jamasurg.2016.4952

[53] Ni X Jia D Chen Y. Is the Enhanced Recovery After Surgery (ERAS) program effective and safe in laparoscopic colorectal cancer surgery? A meta-analysis of randomized controlled trials. J Gastrointest Surg. 2019;23:1502–12. 30859422 10.1007/s11605-019-04170-8

[54] Peng LH Wang WJ Chen J. Implementation of the pre-operative rehabilitation recovery protocol and its effect on the quality of recovery after colorectal surgeries. Chin Med J (Engl). 2021;134:2865–73. 34732661 10.1097/CM9.0000000000001709 PMC8667982

